# The Changing Epidemiology of Breakthrough Invasive Fungal Disease in Allogeneic Hematopoietic Stem Cell Transplant Recipients in the Era of Modified‐Release Posaconazole Prophylaxis

**DOI:** 10.1111/tid.70104

**Published:** 2025-09-26

**Authors:** Shio Yen Tio, Chin Fen Neoh, David Ritchie, Lynette Chee, David C. M. Kong, Leon J. Worth, Michelle K. Yong, Monica A. Slavin

**Affiliations:** ^1^ Department of Infectious Diseases Peter MacCallum Cancer Centre Melbourne Australia; ^2^ National Centre For Infections in Cancer Melbourne Australia; ^3^ Sir Peter MacCallum Department of Oncology University of Melbourne Melbourne Australia; ^4^ Victorian Infectious Diseases Service (VIDS) Royal Melbourne Hospital Melbourne Australia; ^5^ Department of Clinical Haematology Peter Maccallum Cancer Centre The Royal Melbourne Hospital Melbourne Australia; ^6^ Department of Medicine University of Melbourne Melbourne Australia; ^7^ The National Centre for Antimicrobial Stewardship The Peter Doherty Institute for Infections and Immunity Melbourne Australia; ^8^ Faculty of Pharmacy and Pharmaceutical Sciences, Centre For Medicine Use and Safety Monash Institute of Pharmaceutical Sciences Monash University Melbourne Australia

**Keywords:** allogeneic hematopoietic stem cell transplant, breakthrough invasive fungal disease, invasive mould disease, invasive yeast infections, posaconazole prophylaxis

## Abstract

**Background:**

Allogeneic hemopoietic stem cell transplant (aHSCT) recipients are at high‐risk for invasive fungal disease (IFD), even with mould‐active antifungal prophylaxis (AFP).

**Methods:**

This was a retrospective, observational, single‐center cohort study involving 300 adult aHSCT recipients transplanted from January 2017–May 2020. Patient demographics, underlying hematological malignancy (HM), transplant characteristics and AFP were described. The primary objectives were rate and characteristics of breakthrough IFD (bIFD) within 1‐year post‐transplant.

**Results:**

Of 300 aHSCT recipients, 195 (65%) were males; median age at transplantation was 54 years (IQR 43–62). Acute leukemia was the most common underlying HM (50%), and modified‐release posaconazole was the main primary AFP (88%). B‐IFD occurred in 26 patients (9%): 14 breakthrough invasive mould diseases (bIMD), which *Aspergillus* species predominated (56%), followed by *Lomentospora prolificans* (31%); and 12 breakthrough invasive yeast infections, with *Nakaseomyces glabratus* most frequently isolated. Neither *Aspergillus fumigatus* complex nor *Candida albicans* was cultured as breakthrough organisms. bIMD occurred late post aHSCT at median of 167 days, whereas breakthrough invasive yeast infection occurred at median of 21 days. Twelve patients (46%) had therapeutic‐drug‐monitoring at time of bIFD—all were within therapeutic range. All‐cause mortality at 12‐weeks from bIMD and breakthrough invasive yeast infection infections were 50% and 58%, respectively.

**Conclusion:**

Although bIFD rate was consistent with other reports, mortality after bIFD remained significant. Use of mould‐active AFP likely explained the changing epidemiology of fungal isolates. Resistant breakthrough fungal organisms, especially *L. prolificans*, reflected local epidemiology. Ongoing surveillance of IFD including resistant organisms is warranted to optimize treatment and patient outcome.

## Graphical abstract

This single‐center retrospective cohort study on 300 allogeneic hematopoietic stem cell transplant recipients from January 2017 to May 2020, in which posaconazole modified release was the most common primary antifungal prophylaxis, demonstrated a breakthrough invasive fungal disease (bIFD) rate of 9%, with associated significant all‐cause mortality at 12 weeks. Ongoing surveillance of IFD including resistant organisms is warranted to optimize treatment and patient outcome.

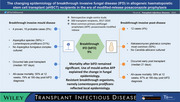

## 1 Introduction

Invasive fungal disease (IFD) remains a leading cause of mortality and morbidity in immunosuppressed patients who undergo allogeneic hematopoietic stem cell transplant (aHSCT). With the introduction of mould‐active antifungal prophylaxis (AFP) as standard‐of‐care, the incidence of IFD has reduced considerably, but a subset of patients develops breakthrough IFDs (bIFDs). Risks for bIFDs include prior antibiotic exposure, biofilm formation on intravenous (IV) catheters, prolonged neutropenia, corticosteroid use, antifungal non‐susceptibility, inappropriate antifungal choices, and dosage resulting in inadequate therapeutic levels [1, 2]. Mortality associated with bIFDs is substantial, with attributable mortality up to 61% [2, 3]. Diagnostic challenges due to reduced sensitivity to various culture and molecular techniques in the setting of AFP, and lack of consensus in its management lead to poor patient outcome [3, 4]. In addition, a shift in epidemiology of bIFDs and emergence of resistant fungal organisms are increasingly reported [5, 6, 7, 8, 9].

Accordingly, not only is the knowledge of local fungal epidemiology crucial in understanding the fungal species contributing to bIFDs, which allows timely initiation of empirical antifungal treatment, it also provides opportunity for development of novel diagnostic tools and trials of new antifungals which may soon be available for clinical use. Neoh et al. conducted an Australian‐wide multicenter retrospective cohort study on non‐*Aspergillus* moulds (NAM) and observed a predominance of *Lomentospora prolificans* and *Scedosporium* spp. infections over species such as Mucorales and *Fusarium* species, in contrast with other regions [10]. These infections, particularly *L. prolificans*, were often associated with high rates of treatment failure and mortality [11].

Given the above, we evaluated the rate and characteristics of bIFDs on AFP. Such a study has not been previously conducted in Australia. Therefore, the objective of the study was to describe bIFDs in a major transplant center in Victoria, Australia, which largely utilized modified‐release (MR) formulation of posaconazole as primary prophylaxis since mid‐2015 when it replaced posaconazole suspension. It is anticipated that this work would inform future diagnostic and therapeutic strategies, leading to improved clinical outcomes in these high‐risk patients.

## 2 Methods

### 2.1 Study Design

This was a retrospective, observational, single‐center cohort study involving 300 consecutive adult aHSCT recipients above 18 years of age undergoing their first allogeneic transplant, from January 2017 to May 2020. Consecutive cases were identified through the hematology transplant recipient database. Consent was waived as the study was considered a quality improvement project. The study obtained Human Research Ethics Committee approval (QA/64470/PMCC‐2020).

Data were collated using a standardized electronic case report form in a secure centralized registry in REDCap. Patient demographics, underlying hematological malignancy (HM), co‐morbidities, transplant characteristics, AFP and complications such as GVHD, respiratory and cytomegalovirus (CMV) infections within a year post‐transplant; outcomes such as intensive care unit (ICU) admission, relapsed disease within a year post‐transplant, and mortality were captured. For those who developed IFD post aHSCT, further information such as microbiology, radiological features, and treatment data were collected. Patients were followed up for at least one year after aHSCT, or until death, whichever occurred first. Patients who developed IFD were followed up for 1 year post diagnosis of IFD.

### 2.2 Transplant Details

The types of transplants, conditioning regimens, donor, and stem cell source were administered according to patients’ underlying HM and local transplant protocol, as was prophylaxis against GVHD. All transplant patients were admitted into single rooms with high‐efficiency particulate air filtration. As part of preventive strategies, hospital‐wide environmental surveillance of invasive mould infections was conducted regularly and patients were encouraged to wear N95 mask post discharge if they encountered construction work.

In accordance with the Australian and local guidelines for AFP [11], posaconazole MR formulation was used as first‐line prophylaxis at our institution for all patients deemed at high risk of IFD (> 10% incidence), based on depth and duration of neutropenia, corticosteroid dose, presence of GVHD, and high risk transplant characteristics. A minority of patients was still receiving posaconazole suspension at time of data collection. Whereas in those whose risk of acquiring IFD are < 5% incidence, namely allogeneic stem cell transplant recipients with expected duration of neutropenia less than 14 days, fluconazole is used. Other AFP agents such as liposomal amphotericin B or voriconazole were used for reasons such as previous IFD prior to transplant. AFP was commenced on Day 0 of aHSCT until 75 days post‐engraftment [12] and continued beyond in the presence of extensive chronic or steroid‐refractory GVHD, ongoing neutropenia or poor engraftment. It was not a standard‐of‐care practice in our center to perform therapeutic drug monitoring (TDM) for patients receiving posaconazole MR formulation due to its reliable oral bioavailability. TDM was however utilized for patients receiving posaconazole suspension and voriconazole. Other routine prophylaxis included trimethoprim–sulfamethoxazole against *Pneumocytis jirovecii* (PJP), initiated from engraftment onwards, and valaciclovir/acyclovir against herpes virus reactivation. No antibacterial prophylaxis was used.

CMV and Epstein–Barr virus (EBV) viral load were monitored twice weekly and a pre‐emptive strategy was employed. IV ganciclovir 5 mg/kg 12‐hourly (or valganciclovir or foscarnet) was administered if clinically significant viral threshold (of approximately 1000 IU/mL) was reached.

### 2.3 Clinical Definitions

Neutrophil engraftment was defined as the time when absolute neutrophil count (ANC) was > 0.5 × 10^9^/L for two consecutive days. Severe neutropenia was defined as ANC < 0.5 × 10^9^/L. Acute and chronic GVHD were classified according to the Glucksberg grading system and National Institutes of Health (NIH) consensus criteria, respectively [13, 14]. We considered significant corticosteroid use as prednisolone dose of > 0.5 mg/kg/day or equivalent for at least 3 weeks. If TDM was performed whilst on mould‐active AFP, the target trough level was > 0.5 mg/L for posaconazole prophylaxis and >1.0 mg/L for voriconazole prophylaxis [15, 16]. These TDM threshold levels are consistent with recommendations by our national guidelines [15]. IFD was sub‐categorized to invasive yeast infections or invasive mould diseases (IMD). Proven or probable IFD was defined according to updated European Organisation for Research and Treatment of Cancer‐Mycoses Study Group Education and Research Consortium (EORTC‐MSGERC) consensus criteria [17].

bIFD was defined in accordance with criteria proposed by MSG‐ERC and the European Confederation of Medical Mycology, i.e. any IFD occurring during exposure to an antifungal drug, including fungi outside the spectrum of activity of an antifungal was regarded as bIFD [18]. The bIFD period covered the time when the antifungal had normally reached steady state and ended at one dosing interval after drug discontinuation. For example, the period of antifungal exposure began 1 day after loading dose of voriconazole was completed and three days after commencement of posaconazole. The period continued until 12 h after the final dose of voriconazole, and 24 h after IV or tablet posaconazole (or 8 h after last dose of posaconazole liquid suspension) [3].

### 2.4 Outcome Measures

The primary objective was to describe the rate and characteristics of bIFD within 1‐year post aHSCT. Secondary objectives included bIFD‐related mortality, factors associated with increased risk of breakthrough invasive yeast infections and IMDs, all‐cause 30‐day, 84‐day (12 weeks), 180‐day, and 1‐year mortality from breakthrough invasive yeast and mould disease. We also sought to evaluate the current practice of AFP use in our center and to describe TDM use, when performed.

### 2.5 Statistics

Descriptive analyses were used to report patient demographics, underlying HM, transplant‐associated complications, details of bIFD and bIFD‐related mortality. Thirty‐day, 84‐day (12 weeks), 180‐day, and 1‐year all‐cause mortality from both breakthrough invasive yeast and mould infections were evaluated. Factors associated with increased risk of breakthrough invasive yeast and IMD respectively, were analyzed using Cox proportional hazards regression. All analyses were performed using Stata version 16·1 (StataCorp LP, College Station, Texas, US), with *p* values < 0.05 considered significant.

## 3 Results

### 3.1 Patient Demographics and Transplant Characteristic

Table 1 summarizes patients’ underlying HM and transplant characteristics of the studied cohort. A total of 100 aHSCT recipients were included for study: 195 males (65%) and 105 (35%) females. Median patient age at time of transplantation was 54 years [interquartile range (IQR) 43–62]. Acute leukemia was the most common underlying HM (50%). Overall, patients were followed up for a median of 752 days (IQR 341–1105, maximum duration of follow‐up was 1889 days).

**TABLE 1:** Patients’ underlying hematological malignancy and transplant characteristics.

	Total number of patients (*n* = 300, 100%)
**Underlying hematological malignancy**	
Acute myeloid leukemia (AML)	107 (36%)
Acute lymphoblastic leukemia (ALL)	37 (12%)
Acute leukemia of unspecified types	6 (2%)
Non‐Hodgkin lymphoma (NHL)	41 (14%)
Myelodysplastic syndrome (MDS)	34 (11%)
Myelofibrosis	23 (8%)
Chronic lymphocytic leukemia (CLL)	11 (4%)
Multiple myeloma	10 (3%)
Aplastic anemia	8 (3%)
Chronic myelomonocytic leukemia (CMML)	6 (2%)
Chronic myeloid leukemia (CML)	5 (1%)
Hodgkin lymphoma	5 (1%)
Others^a^	7 (2%)
**Disease status at transplantation**	
Complete remission	195 (65%)
Partial remission	22 (7%)
Stable disease	34 (11%)
Relapsed disease	6 (2%)
Progressive disease	10 (4%)
Untreated	34 (11%)
**Donor type**	
Matched unrelated	149 (50%)
Matched related/sibling	110 (37%)
Haploidentical	41 (13%)
**Type of transplant**	
Reduced intensity conditioning (RIC)	180 (60%)
Myeloablative conditioning (MAC)	99 (33%)
Non‐myeloablative conditioning (NMA)	21 (7%)
**Conditioning regimen**	
Fludarabine/melphalan	121 (40%)
Fludarabine/cyclophosphamide	49 (16%)
Busulfan/cyclophosphamide Fludarabine/cyclophosphamide/TBI	44 (15%) 24 (8%)
TBI‐VP16	23 (8%)
Cyclophosphamide/TBI	16 (5%)
Fludarabine/busulfan	14 (5%)
Others	9 (3%)
**Stem cell source**	
Peripheral stem cell	280 (93%)
Others (bone marrow, cord)	20 (7%)
**GVHD prophylaxis**	
Thymoglobulin/anti‐thymocyte globulin (ATG) containing regimen	136 (45%) 252 (84%)
Cyclosporin/ methotrexate	
**CMV donor/recipient serostatus**	
D+/R+	132 (44%)
D+/R−	34 (11%)
D−/R+	59 (20%)
D−/R−	75 (25%)
**Patients with prior invasive fungal disease (IFD)** Proven pulmonary mucormycosis and scedosporiosis Probable invasive pulmonary aspergillosis (IPA) Probable pulmonary *Fusarium* species infection Possible invasive mould disease (pulmonary) Pulmonary cryptococcal infection Pulmonary *Trichosporon mycotoxinivorans* infection	19 (6%) 1 9 1 4 3 1

Abbreviation: TBI = total body irradiation; CMV = cytomegalovirus; D = donor; R = recipient.

^a^ Systemic mastocytosis, T‐lymphoblastic lymphoma, chronic granulomatous disease, Sezary syndrome, T‐cell prolymphocytic leukemia, blastic plasmacytoid dendritic cell neoplasm.

Nineteen patients (6%) had prior IFD prior to transplant, 15 of whom had IMD. Eleven of these 15 patients experienced complete symptom and radiological resolution prior to allogeneic stem cell transplant. Four patients experienced stable disease at time of transplant, as defined using the European Organisation for Research and Treatment of Cancer and Mycoses Study Group (EORTC/MSG) clinical trial definitions of response to antifungal therapy in patients with invasive mould disease, and none developed post‐transplant bIFD. As for the remaining four patients with pulmonary *Trichosporon mycotoxinivorans* (*n* = 1) and cryptococcal infections (*n* = 3), three were deemed to have stable disease at time of transplant, and one patient was not assessed. None of these four patients had bIFD post‐transplant. Overall, all these patients with previous IFD were either adequately treated or stable prior to their transplant.

### 3.2 AFP Initiated at Time of Transplantation

A total of 283 patients (94%) were initiated or continued mould‐active AFP at time of transplantation. Posaconazole MR formulation was the drug of choice in 265 patients (88%), of whom 80 patients (30%) had TDM performed; nine patients were administered posaconazole suspension (3%) but only two patients (22%) had TDM, six patients (2%) were administered voriconazole prophylaxis, of whom three patients (50%) had TDM, and three patients (1%) received liposomal amphotericin‐based prophylaxis. The remaining 17 patients (6%) received fluconazole 200 or 400 mg daily. For patients with TDM (*n* = 85), posaconazole and voriconazole drug levels were therapeutic in 74 patients (87%), which ranged 0.51–5.28 mg/L for posaconazole and 1.1–7.3 mg/L for voriconazole, respectively.

### 3.3 Rate and Description of bIFD

There were altogether 31 episodes of IFD observed in 31 patients within the first‐year post aHSCT: 18 cases of proven/probable IMD, 12 cases of invasive yeast infections and 1 case of PJP infection. Of these, 26 cases were breakthrough infections—14 bIMDs and 12 breakthrough invasive yeast infections, equivalent to an overall bIFD rate of 9%. Eighteen patients (69%) were neutropenic prior to bIFD, for median of 21 days (IQR 11–26). Posaconazole MR tablet was the most common AFP used when bIFD occurred (*n* = 22), corresponding to 8% of bIFD rate amongst all patients on this AFP; followed by voriconazole (*n* = 2, 33%), liposomal amphotericin‐B and fluconazole (*n* = 1 each, 33% and 6% respectively). Median duration of AFP prior to breakthrough yeast infections and IMD was 21 days (IQR 17–70) and 102 days (IQR 27–197), respectively. Altogether 12 patients (46%) had TDM performed at time of IFD diagnosis, all of whom had levels within therapeutic range according to the Australian consensus guidelines for optimizing antifungal drug delivery and monitoring (Table 2) [15].

**TABLE 2:** Details of patients with breakthrough invasive fungal disease (bIFD).

	Underlying HM	Prior IFD and status of IFD at transplant	Antifungal prophylaxis (AFP)	Duration of AFP (days)	TDM performed	TDM level (mg/L)	Neutropenia prior to IFD (days)	Breakthrough fungal organisms and classification based on EORTC/MSGERC	Antifungal susceptibility	Directed treatment	Outcome	Overall survival post aHSCT (days)	Overall survival post IFD (days)
**Breakthrough invasive mould diseases**													
1	Non‐Hodgkin lymphoma	Yes, prior IPA. Complete symptom and radiological resolution	Posaconazole tablets	176	Yes	0.76	No	*Aspergillus* species (positive BAL GM 3.51) (probable)	Not applicable	Liposomal amphotericin‐B 5mg/kg/day	Survived	NA	NA
2	AML	No	Posaconazole tablets	241	No	NA	Yes (47 days)	*Lomentospora prolificans* (blood culture) (proven)	Not done	Voriconazole and terbinafine	Died	245	4
3	MDS	No	Posaconazole tablets	281	Yes	0.67	No	*Lomentospora prolificans* (blood culture), *Aspergillus niger* complex (BAL culture, positive BAL GM 6.1) (proven)	***Lomentospora prolificans***: Voriconazole MIC = 8, Itraconazole MIC > 16, Posaconazole MIC > 8, Fluconazole MIC > 256, Amphotericin B MIC > 8, Anidulafungin MIC > 8, 5‐FC MIC > 64 ***Aspergillus niger* complex**: not done	Liposomal amphotericin‐B 5mg/kg/day. Died before treatment for *Lomentospora* spp. infection	Died	288	7
4	CML/CMML	No	Posaconazole tablets	27	Yes	1.09	Yes (21 days)	*Rhizopus microspores* and *Curvularia* species (both detected via PCR from BAL and lung tissue biopsy) (proven)	Not applicable	Liposomal amphotericin‐B 5mg/kg/day and posaconazole	Died	141	114
5	AML	No	Posaconazole tablets	157	No	NA	Yes (16 days)	*Lomentospora prolificans* (sputum culture) (probable)	Not done	Voriconazole and terbinafine	Died	516	359
6	AML	No	Posaconazole tablets	106	No	NA	No	*Aspergillus* species (positive BAL GM 4.9) (probable)	Not applicable	Voriconazole	Died	232	126
7	AML	No	Posaconazole tablets	52	Yes	0.61	Yes (30 days)	*Aspergillus nidulans* complex (positive sino‐nasal culture and PCR on tissue biopsy) (probable)	Anidulafungin MIC = 0.015, Voriconazole MIC = 0.12, Itraconazole MIC = 0.25, Fluconazole MIC = 128, Posaconazole MIC = 0.12, Amphotericin B MIC = 4.0	Liposomal amphotericin‐B 5mg/kg/day and terbinafine, then switched to voriconazole	Died	875	145
8	AL of ambiguous lineage	No	Posaconazole tablets	98	No	NA	No	*Lomentospora prolificans* (BAL culture) (probable)	Not done	Voriconazole and terbinafine	Survived	NA	NA
9	T‐PLL	No	Posaconazole tablets	268	Yes	2.67	No	*Aspergillus* species (positive BAL GM 1.56) (probable)	Not applicable	Liposomal amphotericin‐B 3mg/kg/day	Died	324	56
10	AML **(Previous IPA)**	Yes, prior IPA. Complete symptom and radiological resolution	Posaconazole tablets	197	Yes	2.04	No	*Aspergillus* species (positive BAL GM 1.35) (probable)	Not applicable	Voriconazole	Died	229	32
11	CML/CMML	No	Posaconazole tablets	9	No	NA	Yes (11 days)	*Lomentospora prolificans* (blood culture) (proven)	Not done	Died before treatment	Died	11	2
12	AML	No	Liposomal amphotericin‐B 100mg thrice weekly	26	No	NA	Yes (29 days)	*Aspergillus nidulans* (positive BAL GM 6.6 and *Aspergillus nidulans* PCR on BAL) (probable)	Not applicable	Voriconazole	Died	1638	14
13	AML	No	Voriconazole	63	Yes	1.3	Yes (61 days)	*Aspergillus* species (positive BAL GM 18.65) (probable)	Not applicable	Liposomal amphotericin‐B 3mg/kg/day	Died	199	136
14	AML **(Previous possible IMD)**	Yes, prior possible IMD. Complete symptom and radiological resolution	Voriconazole	9	No	NA	Yes (9 days)	*Aspergillus* species (positive BAL GM 3.56) (probable)	Not applicable	Echinocandin	Died	43	34
**Breakthrough invasive yeast infections**													
15	CLL	No	Posaconazole tablets	166	No	NA	No	*Nakaseomyces glabratus* complex (proven)	Anidulafungin MIC = 0.03, Voriconazole MIC = 8, Itraconazole MIC > 16, Fluconazole MIC > 256, Amphotericin B MIC = 1.0	Echinocandin	Died	221	55
16	MDS	No	Posaconazole tablets	22	No	NA	Yes (22 days)	*Saccharomyces cerevisiae* (proven)	Not available	Echinocandin	Died	35	13
17	MDS	No	Posaconazole tablets	114	No	NA	No	*Candida parasilopsis* (proven)	Not available	Echinocandin	Died	169	55
18	CLL	No	Posaconazole tablets	12	No	NA	Yes (10 days)	*Nakaseomyces glabratus* complex (proven)	Anidulafungin MIC = 0.03, Voriconazole MIC = 2, Itraconazole MIC > 16, Fluconazole MIC > 64, Amphotericin B MIC = 2.0	Echinocandin	Survived	NA	NA
19	Myelofibrosis	No	Posaconazole tablets	27	Yes	0.51	Yes (24 days)	Mixed *Meyerozyma guilliermondii* and *Nakaseomyces glabratus complex* (proven)	***Meyerozyma guilliermondii***: Anidulafungin MIC = 1.0, Voriconazole MIC = 0.12, Fluconazole MIC = 4.0, Amphotericin B MIC = 0.25 ***Nakaseomyces glabratus* complex**: Anidulafungin MIC = 0.03, Voriconazole MIC = 1.0, Fluconazole MIC = 128, Amphotericin B MIC = 1.0	Echinocandin	Died	168	141
20	MDS	No	Posaconazole tablets	18	Yes	1.44	Yes (21 days)	*Nakaseomyces glabratus* complex (proven)	Anidulafungin MIC = 0.03, Voriconazole MIC = 0.25, Itraconazole MIC = 0.5, Fluconazole MIC = 8.0, posaconazole MIC = 1.0, Amphotericin B MIC = 0.5	Echinocandin	Survived	NA	NA
21	Aplastic anemia	No	Posaconazole tablets	4	No	NA	Yes (6 days)	*Issatchenkia orientalis* (now known as *Pichia kudriavzevii*) (proven)	Not done	Died before treatment	Died	4	0
22	ALL	No	Posaconazole tablets	20	Yes	1.87	Yes (20 days)	Mixed *Meyerozyma guillermondii* and *Saccharomyces cerevisiae* (proven)	***Meyerozyma guilliermondii***: Anidulafungin MIC = 0.5, Voriconazole MIC = 1.0, Fluconazole MIC = 16, Itraconazole MIC > 16, Amphotericin‐B MIC = 0.5 ***Saccharomyces cerevisiae***: Not done as no CLSI guidelines exist for the interpretation of MICs for this organism	Echinocandin	Died	38	18
23	Hodgkin lymphoma	No	Posaconazole tablets	133	Yes	1.39	Yes (2 days)	*Nakaseomyces glabratus* complex (proven)	Anidulafungin MIC = 0.06, Voriconazole MIC = 2.0, Itraconazole MIC = 1.0, Fluconazole MIC > 128, Amphotericin B MIC = 0.5	Echinocandin	Died	640	65
24	AML	No	Posaconazole tablets	17	No	NA	Yes (26 days)	*Saccharomyces cerevisiae* (proven)	Not done as no CLSI guidelines exist for the interpretation of MICs for this organism	Echinocandin	Died	26	9
25	CML/CMML	No	Posaconazole tablets	17	Yes	0.77	Yes (17 days)	*Nakaseomyces glabratus* complex (proven)	Anidulafungin MIC = 0.03, Voriconazole MIC = 0.5, Itraconazole MIC = 1.0, Fluconazole MIC = 16, Amphotericin B MIC = 1.0	Echinocandin	Survived	NA	NA
26	Myelofibrosis	No	Fluconazole tablets	27	No	NA	Yes (26 days)	*Pichia kudriavzevii* (proven)	Anidulafungin MIC = 0.12, Voriconazole MIC = 0.5, Itraconazole MIC = 0.5, Fluconazole MIC = 64, Amphotericin B MIC = 1.0	Echinocandin	Survived	NA	NA

Abbreviations: HM = hematological malignancy; TDM = therapeutic drug monitoring; IFD = invasive fungal disease; aHSCT = allogeneic hematopoietic stem cell transplant; IPA = invasive pulmonary aspergillosis; NA = not applicable; BAL = bronchoalveolar lavage; GM = galactomannan; AML = acute myeloid leukemia; MDS = myelodysplasia; MIC = minimum inhibitory concentration; CML/CMML = chronic myeloid leukemia/ chronic myelomonocytic leukemia; IMD = invasive mould disease; CLL = chronic lymphocytic leukemia; ALL = acute lymphocytic leukemia; EORTC/MSGERC = European Organisation for Research and Treatment of Cancer‐Mycoses Study Group Education and Research Consortium.

#### 3.3.1 Characteristics of Breakthrough Invasive Mould Disease (bIMDs), Associated Risk Factors, and Mortality

There were 4 proven and 10 probable bIMDs, equivalent to rate of 5%, of which two proven cases were mixed mould infections (Table 2). Three patients had prior IMD prior to breakthrough probable invasive pulmonary aspergillosis (IPA). Where the mould organisms were identified for proven/probable infections (*n* = 16), *Aspergillus* species predominated (*n* = 9, 56%), followed by *L. prolificans* (*n* = 5, 31%), and one each of Mucorales and *Curvularia* species (*n* = 1 each, 6%). Amongst the *Aspergillus* species, the majority (*n* = 7) were diagnosed via non‐culture‐based method (positive *Aspergillus nidulans* PCR and/or galactomannan [GM]), and two *Aspergillus* species were cultured (*Aspergillus niger* complex and *A. nidulans* complex, each *n* = 1). Eight patients had positive GM optical density index (ODI) as per EORTC‐MSGERC criteria [17] in bronchoalveolar lavage (BAL). Median ODI of BAL GM was 4.23 (range 1.35–18.65). Serum GM was performed in only two patients, and both were negative. Of note, there were no *Aspergillus fumigatus* complex cultured as breakthrough organisms (Table 2).

Pneumonia was the most common clinical presentation (*n* = 10), followed by disseminated infections (*n* = 3) and infection of the sino‐nasal region (*n* = 1). Median duration from transplant to diagnosis of bIMD was 167 days (IQR 63–268).

Due to the low number of events, only univariate analysis was performed to determine the factors which increased the risk of bIMDs at 180‐day from aHSCT (as aHSCT to bIMDs occurred at median of 167 days). Table 3 summarizes these potential variables. Previously treated IMD, significant corticosteroid use, relapse and non‐relapse causes of severe neutropenia prior to bIMDs, ICU admission and relapsed disease within 180 days post‐transplant were all statistically significant predictors in univariate analysis. Interestingly, neither grade II‐IV acute GVHD nor moderate to severe chronic GVHD was significantly associated with increased bIMDs.

**TABLE 3:** Univariate analysis of factors associated with increased risk of breakthrough invasive mould diseases (bIMD) at 180 days following allogeneic hematopoietic stem cell transplant.

Variables	Univariate analysis		
	Hazards ratio	Confidence interval	*p* value
Sex	0.79	0.24–2.53	0.696
Age at time of aHSCT	0.99	0.95–1.03	0.722
Previous IMD	5.93	1.65–21.27	0.006
Grade II–IV aGVHD	1.46	0.46–4.68	0.517
Moderate to severe cGVHD	1.16	0.36–3.72	0.792
Significant corticosteroid use	5.05	1.41–18.13	0.013
CMV viremia needing treatment	1.06	0.35–3.19	0.904
Respiratory viral infection	1.45	0.45–4.62	0.529
Severe neutropenia prior to bIMD (overall)	31.10	10.77–89.77	<0.001
Relapse causes of severe neutropenia prior to bIMD	9.93	1.29–75.97	0.027
Non‐relapse causes of severe neutropenia prior to bIMD	28.97	10.13–82.79	<0.001
ICU admission	21.44	4.79–95.89	<0.001
Relapsed disease within 180‐days from aHSCT	3.94	1.09–14.20	0.036

Abbreviations: aGVHD = acute graft‐versus‐host‐disease; aHSCT = allogeneic hematopoietic stem cell transplant; cGVHD = chronic graft‐versus‐host‐disease; CMV = cytomegalovirus; ICU = intensive care unit; IFD = invasive fungal disease; IMD = invasive mould disease.

From diagnosis of bIMD, all‐cause 180‐day mortality was 79% (*n* = 11), and median survival was 34 days (IQR 7–126). Thirty‐day, 84‐day (12 weeks), and 1‐year all‐cause mortality post‐bIMD diagnosis were 29% (*n* = 4), 50% (*n* = 7), and 86% (*n* = 12), respectively.

Five deaths were directly attributed to bIMD (36%): three disseminated *L. prolificans* infections (one co‐infection with invasive pulmonary *A. niger*), one proven invasive pulmonary *Mucorales* and *Curvularia* co‐infection and one probable IPA. All patients with disseminated *L. prolificans* infections died within a week of bIMD diagnosis.

#### 3.3.2 Characteristics of Breakthrough Invasive Yeast Infections, Associated Risk Factors and Mortality

Twelve patients developed breakthrough invasive yeast infections (rate of 4%) with 14 yeast species isolated. Of these 12 cases, 11 had yeast organisms isolated from blood culture, and the remaining case was an invasive candidiasis from an intra‐abdominal/retroperitoneal collection. *Candida* species was the most common breakthrough yeast isolate. The yeast species isolated were *Nakaseomyces glabratus* complex (previously known as *Candida glabrata*, *n* = 5), *Saccharomyces cerevisiae* (*n* = 2), *Pichia kudriavzevii (*previously *Candida krusei)* (*n* = 2)*, Candida parapsilosis* complex (*n* = 1), a case each of mixed *Meyerozyma guillermondii* (previously *Candida guillermondii*) and *N. glabratus* complex (*n* = 1) and mixed *Meyerozyma guillermondii* and *Saccharomyces cerevisiae* (*n* = 1) (Table 2). Of note, there was no *Candida albicans* isolated. Median duration from aHSCT to diagnosis of breakthrough invasive yeast infection was 21 days (IQR 17–70).

Minimum inhibitory concentration (MIC) for various antifungals was available for 9 of 14 yeast species, including 6 *N. glabratus* complex, of which according to CLSI epidemiological cutoff values (ECVs), 5 were considered non‐wild type (NWT) for voriconazole and 2 were NWT for itraconazole [19] (Table 2).

On univariate analysis, increased risk of breakthrough invasive yeast infections at 30‐day from aHSCT (given median duration from aHSCT to breakthrough yeast infection was 21 days) was associated with severe neutropenia (*p* < 0.001) and ICU admission (*p* = 0.004) (Table 4).

**TABLE 4:** Univariate analysis of factors associated with increased risk of breakthrough invasive yeast infections at 30‐day from allogeneic hematopoietic stem cell transplant.

Variables	Hazards ratio	Confidence interval	*p* value
Sex	2.65	0.84–8.36	0.096
Age at time of aHSCT	1.01	0.96–1.05	0.630
Other stem cell source (bone marrow or cord blood)	1.28	0.16–9.93	0.812
Grade 3–4 mucositis	1.53	0.46–5.11	0.481
TPN	2.38	0.64–8.78	0.194
Grade II–IV aGVHD	1.80	0.48–6.65	0.377
Severe neutropenia prior to breakthrough invasive yeast infections	89.19	19.54–407.14	< 0.001
ICU admission	5.87	1.77–19.51	0.004

Abbreviations: aGVHD = acute graft‐versus‐host‐disease; aHSCT = allogeneic hematopoietic stem cell transplant; ICU = intensive care unit.

Thirty‐day all‐cause mortality after the diagnosis of breakthrough invasive yeast infection was 33% (*n* = 4), with overall median survival of 11 days (IQR 4–15) post diagnosis of breakthrough yeast infection, whereas 84‐day (12 weeks), and 180‐day mortality were 58% (*n* = 7) and 67% (*n* = 8), respectively (1‐year mortality was similar to 180‐day mortality). One yeast infection‐related mortality was attributable to invasive *Issatchenkia orientalis* (now known as *Pichia kudriavzevii*) infection in a patient who died 4 days after stem cell infusion.

### 3.4 Remaining IFD That Were Non‐Breakthrough Infections

There were four probable IPA: two had positive culture for *A. fumigatus* complex (one case also had a positive GM ODI of 2.876 and itself was a mixed infection with *Chaetomium atrobrunneum*), and the other two cases were diagnosed via positive BAL GM (ODI 1.11 and 4. 65). These four patients already had their AFP discontinued prior to development for IPA, which occurred at a median of 437 days (IQR 258–646) post aHSCT. Two patients were treated with voriconazole and the other two with posaconazole, and all of them had at least 90 days of treatment. All were still alive at 1‐year post diagnosis of IFD.

## 4 Discussion

We report a contemporary cohort of 300 aHSCT recipients, the majority (88%) of whom were receiving posaconazole MR formulation for AFP. This study has provided important insights into the local epidemiology and characteristics of bIFD, summarized in Table 5. We observed a bIFD rate of 9% (5% for bIMD, 4% for breakthrough yeast infections) with high all‐cause mortality rates. We also noted a change in fungal epidemiology, in which no *A. fumigatus* complex or *C. albicans* were cultured as bIFDs. Furthermore, we found that *L. prolificans* infection (a mould resistant to all available antifungals) was the most common breakthrough NAM infection in our cohort, in contrast with findings from a systematic review by Boutin et al. where Mucorales was second after breakthrough invasive aspergillosis (IA) [3]. The distinct fungal epidemiology, timing of infection and antifungal susceptibility observed with bIMD and breakthrough invasive yeast infections will raise clinicians’ awareness to screen for bIFD and to initiate appropriate management in a timely fashion. This information will also inform future diagnostic and therapeutic strategies that aims to improve clinical outcomes. It is well‐recognized that *L. prolificans* is intrinsically resistant to many antifungals and fungemia with this organism is associated with high mortality rates [11], as demonstrated in our study in which three patients with disseminated infections died within 1 week of diagnosis, highlighting the urgent need for antifungal agents active against this pathogen. This knowledge of local fungal epidemiology equips clinicians to source alternative antifungal promptly such as Olorofim, a novel antifungal in the pipeline that has shown promising therapeutic effect against this multidrug‐resistant fungi [11, 20].

**TABLE 5:** Summary of findings for breakthrough invasive mould disease (IMD) and breakthrough invasive yeast infections.

	Breakthrough invasive mould disease (bIMD)	Breakthrough invasive yeast infections
Number of cases (*n*, rate in %)	14 (5%)	12 (4%)
Fungal isolates most frequently isolated (*n*, %)	*Aspergillus* species (*n* = 9, 56%) > *Lomentospora prolificans* (*n* = 5, 31%) No *Aspergillus fumigatus* complex cultured	*Nakaseomyces glabratus* complex (*n* = 6, 50%) No *Candida albicans* cultured
Duration from aHSCT to bIFD (median, IQR)	167 days (IQR 63–268)	21 days (IQR 17–70)
Number of patients with severe neutropenia prior to diagnosis of bIFD (%)	8 (57%)	10 (83%)
Duration of neutropenia (median, IQR)	25 days (IQR 13–38)	20 days (IQR 10–24)
Types of AFP (*n*, %)	Posaconazole MR tablets (*n* = 11, 79%), voriconazole (*n* = 2, 14%), liposomal amphotericin B (*n* = 1, 7%)	Posaconazole MR tablets (*n* = 12, 100%)
Duration of AFP prior to bIFD (median, IQR)	102 days (IQR 27–197)	21 days (IQR 17–70)
Number of patients with TDM (*n*, %)	7 (50%), all within therapeutic levels	5 (42%), all within therapeutic levels
Risk factors based on univariate analysis	Previously treated IMD, significant corticosteroid use, relapse, and non‐relapse causes of severe neutropenia prior to bIMD, ICU admission and relapsed disease within 180 days post‐transplant	Severe neutropenia prior to breakthrough invasive yeast infection, ICU admission
All‐cause mortality at 12 weeks post diagnosis of bIFD (*n*, %)	7 (50%)	7 (58%)
Duration of survival post diagnosis of bIFD (median, IQR)	34 days (IQR 7–126)	36 days (IQR 11–60)
Attributable death (*n*, %)	5 (36%)	1 (8%)

Abbreviations: AFP = antifungal prophylaxis; aHSCT = allogeneic hemopoietic stem cell transplant; bIFD = breakthrough invasive fungal disease; bIMD = breakthrough invasive mould disease; ICU = intensive care unit; IQR = interquartile range; MR = modified release; TDM = therapeutic drug monitoring.

Whilst IA was the most common bIMD accounting for 56% of all moulds isolated (*n* = 9), all the *Aspergillus* species identified were non‐fumigatus *Aspergillus* species (two *A. nidulans* complex and one *A. niger* complex), in contrast with the *Aspergillus* species identified in non‐breakthrough IMD (two *A. fumigatus* complex). Although Mucorales were more frequently diagnosed in an earlier study [21], an updated study recently performed from 2016–2023 observed that *Scedosporium/Lomentospora* species were now more prevalent than Mucorales in our region, accounting for 41% of all NAM infections compared to 31% of episodes of Mucorales infections in our region [10]. Widespread use of triazoles, especially posaconazole has likely driven the shift towards non‐fumigatus *Aspergillus* species and the change in local NAM breakthrough organisms [8, 22], given that breakthrough Mucorales infections are usually associated with voriconazole use [3].

Breakthrough IMD occurred late post‐transplant, often at 100 days post‐transplant or later [23, 24], as shown herein (median of 167 days, range 9–1624 days). This could be explained by evolution in transplant procedures, namely the use of reduced intensity conditioning procedures, frequent use of peripheral stem cells source and prolonged administration of mould‐active AFP [25]. Risk factors for development of bIMD included previous proven/probable IMD, significant corticosteroid use, severe neutropenia prior to bIMD, ICU admission, and relapsed disease within 180‐day post aHSCT, these findings being consistent with previous studies [26, 27, 28]. Acute or chronic GVHD were not predictors of bIMD despite significant corticosteroid use as one of the risk factors. In‐depth review of patients’ data revealed that significant corticosteroid use was also applied in non‐GVHD related conditions such as post‐transplant respiratory failure of unclear cause.

Given late occurrence of bIMD, some studies have proposed longer duration of AFP for months or even years post‐transplant [24]. In our series of bIMDs, all patients were on prior mould‐active AFP for median of 102 days, suggesting that host factors such as primary graft failure and relapsed disease with secondary neutropenia potentially contributed to acquisition of IMD, as shown in our analysis. Under‐recognition of late bIMD could be another reason for those in our cohort who developed bIMD and died approximately within a month post diagnosis. Indeed, our observations suggest the need to be vigilant for the development of bIMD late in the course of transplant.

In distinction to bIMD, it is important to highlight that breakthrough invasive yeast infections occurred early within the first month post‐transplant in our cohort, in contrast with previous studies which demonstrated much later occurrence [23, 29]. Severe neutropenia and ICU admission were identified as the predictors of breakthrough invasive yeast infections in our study. Being critically unwell with severe neutropenia, disruption to mucous membranes, frequent use of broad‐spectrum antibiotics and presence of central venous catheter in the pre‐engraftment and early engraftment period [26, 30, 31] likely explained the above observation. Mucositis and TPN use were not shown to be significant risk factors here, likely secondary to underpowered study.

Amongst the yeast species, *N. glabratus* complex was most frequently isolated and no *C. albicans* was identified. In addition, 5 *N. glabratus* complex harbored higher CLSI ECVs for voriconazole and were considered NWTs for this antifungal. This change in spectrum is well‐recognized due to extensive use of azole prophylaxis [9, 23, 29, 31, 32, 33]; in fact Ostrosky‐Zeichner et al. found that *N. glabratus* was the most common breakthrough pathogen associated with posaconazole use [34], consistent with our findings.

Another notable finding in this study was the significant all‐cause mortality post diagnosis of bIFD: 50% and 58% at 12 weeks, 79% and 67% at 180‐day for bIMD and breakthrough invasive yeast infections, respectively, and attributable death from bIMD was also significant at 36%. Therefore, mitigation strategies are needed to prevent or reduce IFD from the outset. Aseptic technique and central line care bundle [35], judicious use of antimicrobials [36], strict infection control and surveillance in the setting of rising azole‐resistant *Aspergillus* species and resistant *Candida* species like *Candida auris* [37, 38] are important to prevent IFD. Early bronchoscopy in patients who develop pulmonary infiltrates [39], and development of novel molecular tools such as specific *Lomentospora/Scedosporium* species and Mucorales PCR [40] are vital in enhancing diagnosis and identification of IFD. In addition, there is also a need for careful TDM in selected patients such as those with severe gut GVHD or obesity. In our cohort, although only 46% of our patients had TDM requested at time of bIFD diagnosis, the levels were all within therapeutic range suggesting that other factors such as high fungal inoculum and patient's net immunosuppression state played equally important roles in acquisition of IFD [41]. It was unclear if the remaining 54% of bIFD could have been due to suboptimal antifungal level. Furthermore, for those on posaconazole suspension and voriconazole in which TDM is recommended, only 20% and 50% of patients had TDM performed, respectively, although the number of patients on these agents was small (total *n* = 16). Antifungal stewardship is hence needed in this population to optimize antifungal use and to reduce toxicity of antifungals [42, 43], including monitoring TDM when appropriate.

Our study was limited by its retrospective nature and due to the low number of IFD, multivariate analysis could not be performed for either breakthrough invasive yeast infections and bIMD and potentially missed confounding predictors. We did not capture pre‐transplantation patient factors such as the hematopoietic cell transplantation‐specific comorbidity index (HCT‐CI) and performance status (PS), post‐transplantation factors e.g. maintenance therapy such as Venetoclax or hypomethylating agent, the type and duration of antibiotics use prior to IFD and antifungal susceptibilities of different mould and yeast species cultured, as all these could also have an impact on the incidence of bIFD [31, 36]. Nonetheless we reported real‐world experience related to the current practice of AFP use in our center, usage of TDM, and associated rates of bIFD, which has not been previously described locally.

## 5 Conclusion

This study reported an overall bIFD of 9% in a transplant center which largely utilized posaconazole MR tablets as primary AFP, and this was associated with significant mortality post diagnosis of bIFD. The changing epidemiology of fungal isolates in the setting of mould‐active AFP was confirmed. Ongoing surveillance of IFD, especially azole‐resistant *Aspergillus* species and resistant fungal organisms that are locally prevalent such as *L. prolificans*, enhanced diagnostic tests and access to newer antifungals active against resistant moulds, and attention to antifungal stewardship in this cohort of patients is needed to optimize patient outcome.

## Author Contributions

Monica A. Slavin, Michelle K. Yong and Shio Yen Tio conceived the study. Data was collected by Shio Yen Tio and research assistants listed below under “Acknowledgment”. Data cross‐check and analysis was done by Shio Yen Tio, with results evaluated by Michelle K. Yong, Leon J. Worth and Monica A. Slavin. Draft manuscript was prepared and edited by Shio Yen Tio, with comments provided by all authors. All authors reviewed the final version of manuscript.

## Conflicts of Interest

This is an investigator‐initiated study supported by MSD Australia, where Monica A. Slavin is the principal investigator, but it has no role in study design, data collection, data analysis, data interpretation, or writing of the report. All other authors declared no conflict of interest related to this work.

## Disclosure

ICMJE disclosure form for each authors are attached for perusal.
